# Medium-specific escape motivation in internet gaming disorder and problematic social media use among Chinese university students: the role of self-control

**DOI:** 10.3389/fpubh.2026.1896731

**Published:** 2026-08-26

**Authors:** Zinan Wang, Lihui Ren, Ruoyu Zhou, Limin Wu, Yuan Liang, Ying Zhang, Yirui Wang

**Affiliations:** School of Future Education, Qingdao Hengxing University of Science and Technology, Qingdao, China

**Keywords:** compensatory internet use, escape motivation, internet gaming disorder, medium specificity, problematic social media use, self-control

## Abstract

**Background:**

Escape motivation is a well-established correlate of problematic technology use, but it is commonly measured as a general tendency. This cross-sectional study examined whether escape motivation tied to a particular medium shows differential associations with problems in that medium and examined the role of self-control.

**Methods:**

A total of 358 Chinese university students (232 women; mean age 20.2 years) completed measures of game and short-video escape motivation, self-control, internet gaming disorder (IGD) symptoms, problematic social media use (PSMU), and relevant covariates. The primary regression model simultaneously entered both escape motivations and adjusted for sex, age, and daily gaming time; minimally adjusted and stringent models were treated as sensitivity analyses.

**Results:**

In the primary model, game escape motivation was associated with IGD symptoms (*β* = 0.34, *p* < 0.001), whereas short-video escape motivation was not (*β* = 0.01, *p* = 0.872); the coefficient difference was significant (Wald *p* = 0.015). Short-video escape motivation was associated with PSMU (*β* = 0.31, *p* < 0.001), whereas game escape motivation was not (*β* = 0.09, *p* = 0.216), but the coefficient-difference test was non-significant (Wald *p* = 0.133). The short-video association weakened to non-significance under stringent adjustment. Self-control was negatively associated with both outcomes, while moderation evidence was limited and exploratory.

**Conclusion:**

The game escape–IGD association was robust, whereas the short-video escape–PSMU association remains preliminary. These cross-sectional results do not establish causal, screening, or preventive effects.

## 1 Introduction

Problematic technology use among young people, including internet gaming disorder (IGD) and problematic social media use (PSMU), has become a salient public-health concern. Throughout this article, ‘problematic technology use’ is used as an umbrella descriptor for problematic engagement with digital media; the two specific constructs examined are IGD and PSMU. PSMU is treated here as a non-diagnostic pattern of problematic engagement with social media, not as a formally recognized behavioral addiction; gaming disorder, by contrast, is formally recognized in the ICD-11. IGD is included in the DSM-5 as a condition for further study (1, 2). Across adolescent and adult samples, problematic gaming and social media use have been associated with psychiatric symptoms and functional impairment (3, 4). Identifying the motivational correlates of these behaviors is therefore a relevant public-health research question. Gaming and social media frequently co-occur. Escape from stress or negative affect is a prominent motive in problematic gaming, and compensatory-use accounts more broadly conceptualize problematic online engagement as a response to offline difficulties and unmet needs (5–7).

Among the motives implicated in problematic technology use, escape motivation is one of the most consistently reported. Escape, the use of an activity to avoid or relieve real-life difficulties, is robustly associated with IGD across cultures and is often described as the motive that most strongly distinguishes problematic from non-problematic engagement (5, 6). Within the compensatory internet use framework, problematic online behavior is conceptualized not as a primary pathology but as an attempt to cope with unmet psychological needs and offline stressors (7). From this perspective, escape motivation is a proximal psychological correlate linking distal vulnerability to problematic use.

Despite extensive evidence, escape motivation has typically been studied in relation to a single medium, most often gaming, and treated as a relatively general avoidance tendency. This leaves an important theoretical question unanswered: is escape motivation channel-specific? That is, does escape motivation directed toward a particular medium relate more strongly to problematic use of that same medium, or does a general escape disposition relate similarly to problematic use across media? These alternatives correspond to different measurement interpretations. Evidence of channel specificity would support the use of medium-matched measures in future research, whereas evidence of a general disposition would favor a global escape measure.

Contemporary young people in China engage heavily with online gaming and short-video platforms, two digital activities that differ in their interaction structures and reinforcement patterns. Short-video feeds are characterized by rapid, low-effort consumption and algorithmic personalization (8, 9). Short-video applications are among the most widely used internet services in China, reaching 93.8% of internet users by December 2024 (10), which is why short-video escape was selected as the social-media-side motivation. At the same time, because the outcome measure available in this study (the BSMAS) indexes social media use broadly rather than short-video use specifically, the mapping from short-video escape to PSMU is imperfect. Because these activities afford different experiences, escape motivation oriented toward one may not translate into problematic use of the other. Examining game escape motivation and short-video escape motivation simultaneously, in relation to both IGD symptoms and PSMU, provides a direct test of medium specificity. If each escape motivation were uniquely associated with problems in its own medium but not the other, this would support the view that escape motivation is medium-specific rather than a single general disposition.

A second question concerns whether self-control is negatively associated with escape-related problematic use. Self-control, the capacity to regulate impulses and align behavior with longer-term goals, is broadly associated with better adjustment and less psychopathology (11), and meta-analytic evidence links lower self-control to problematic internet use among students (12). Beyond this main association, self-control may moderate the association between escape motivation and problematic use: individuals with higher self-control may report escape motivation without showing equally high problematic-use scores. Testing moderation therefore examines whether the escape-problem association varies according to self-control.

Finally, the role of family factors in problematic technology use may change with development. Systematic and meta-analytic evidence links warmer and more supportive family environments with lower problematic internet use and IGD among adolescents (13, 14). Emerging adults are navigating a developmental period defined by increased autonomy and self-direction (15), during which the direct association of current family factors may attenuate and individual-level motivational and regulatory processes may become more proximal correlates of problematic use. University students therefore offer an informative developmental context in which to test whether escape motivation and self-control, rather than current family factors, are the salient correlates of problematic technology use.

The present study addressed these questions in a sample of Chinese university students. We tested the following hypotheses:

*H1* (medium specificity): When game escape motivation and short-video escape motivation are entered simultaneously, game escape motivation will be more strongly associated with IGD symptoms, whereas short-video escape motivation will be more strongly associated with PSMU.

*H2* (negative association): Self-control will be negatively associated with both IGD symptoms and PSMU.

*H3* (moderation, exploratory): Self-control may moderate the association between escape motivation and problematic use, such that the association is weaker at higher levels of self-control. Given the number of interactions tested, this hypothesis is treated as exploratory.

As a contextual analysis, we also examined whether current family factors were directly associated with problematic use in this emerging-adult sample.

## 2 Materials and methods

### 2.1 Participants and procedure

Participants were 358 university students (232 women, 126 men; mean age = 20.2 years, SD = 1.4, range 18–25) enrolled at a single university in Shandong Province, China. By year of study, 169 were in Year 1, 81 in Year 2, and 108 in Year 3 or 4. Data were collected through an online questionnaire in 2026. After providing informed consent, participants completed the measures described below.

Data were drawn from a cross-sectional survey project (Ethics No. QHU-FEE-LS [2026] No. 001). The de-identified dataset and codebook are available on the Open Science Framework^1^.

From an initial pool of 380 university responses, 22 cases were removed: 7 for a completion time under 100 s (a threshold below which careful reading of all items is implausible given the questionnaire length), 4 for out-of-range or logically inconsistent responses, 2 for reported age above 30 years (outside the target emerging-adult range), and 9 for near-zero response variance across items (SD < 0.3, indicating non-differentiated straight-line responding). This yielded the analytic sample of 358. Because 24.6% of participants (n = 88) reported not playing games, all gaming-related analyses were repeated in a sensitivity sample restricted to self-reported gamers (*n* = 270), and daily gaming time was included as a covariate in the main models (see Section 2.3 and 3.5).

### 2.2 Measures

#### 2.2.1 Game escape motivation

Four items adapted from the escape subscale of the Motives for Online Gaming Questionnaire (MOGQ) (5), with the Chinese version validated by Wu et al. (16), assessed playing games to escape or relieve real-life difficulties, rated on a 5-point scale. Internal consistency was excellent (*α* = 0.947).

#### 2.2.2 Short-video escape motivation

Four items were adapted from the escape subscale of the validated Chinese version of the MOGQ (5, 16). Because the source items were already available in Chinese, no additional translation or back-translation was undertaken. The adaptation was limited to replacing the activity context of “playing games” with “watching short videos,” while retaining the remaining item content and the 5-point response format. Two bilingual members of the research team independently reviewed the modified wording for semantic equivalence and contextual appropriateness, and differences were resolved through discussion. No formal expert-panel content-validity rating, pilot study, or cognitive interviewing was conducted before data collection; the content validity and respondent interpretation of the adapted measure should therefore be regarded as provisional. Internal consistency was high (*α* = 0.949). The exact administered Chinese wording, corresponding English glosses, and parallel game-escape items are provided in Supplementary Table S1. The factor structure, convergent validity, and discriminant validity of the adapted measure were examined in the present sample (Section 3.2).

#### 2.2.3 Self-control

The 13-item Chinese Self-Control Scale (SCS-C) (17) assessed trait self-control on a 5-point scale; nine items (2–5, 7, 9–11, 13) were reverse-scored, with higher totals indicating greater self-control (*α* = 0.752).

#### 2.2.4 Internet gaming disorder symptoms

IGD symptoms were assessed with an 11-item locally adapted measure based on the Gaming Disorder Scale for Adolescents (GADIS-A) (18), with the Chinese version validated by Chen et al. (19). Although originally developed for adolescents, the Chinese GADIS-A has been validated in samples of Chinese emerging adults, including university students (19), supporting its use here. Items were rated on a 5-point scale (*α* = 0.973).

#### 2.2.5 Problematic social media use

The six-item Bergen Social Media Addiction Scale (BSMAS) (4) assessed PSMU on a 5-point scale (*α* = 0.943). Proposed BSMAS thresholds vary across studies (20, 21). A score of 24 or above, derived from clinical interviews with Chinese adolescents (21), was used solely as a descriptive indicator of higher symptom load. Because this threshold has not been clinically validated in university students, it was not treated as a diagnostic or prevalence criterion.

#### 2.2.6 Covariates

Sex and age were included as covariates. Family factors (parental emotional warmth, parental autonomy support, and parent–child relationship quality) and basic psychological need satisfaction were measured for the contextual analysis. Parental emotional warmth was assessed with the Chinese short-form EMBU (22); basic psychological need satisfaction was assessed with items adapted from the satisfaction subscales of the cross-culturally validated need scale by Chen et al. (23), comprising 12 items (four per need: autonomy, competence, relatedness) rated on a 5-point scale. Depressive and anxiety symptoms were screened with the PHQ-2 (24) and GAD-2 (25).

### 2.3 Statistical analysis

Analyses were conducted in Python (NumPy, SciPy, statsmodels, scikit-learn) and R (lavaan). We first examined reliability, descriptive statistics, common method bias (Harman’s single-factor test supplemented by a confirmatory single-factor sensitivity model; Section 3.2), and bivariate correlations. The adapted short-video escape measure was evaluated through an initial principal-component analysis followed by maximum-likelihood confirmatory factor analyses comparing one- and two-factor structures across the eight escape items, average variance extracted (AVE), composite reliability (CR), and the heterotrait–monotrait ratio (HTMT). To test medium specificity, game and short-video escape motivation were entered simultaneously as focal explanatory variables for IGD symptoms and PSMU. Model B was designated the primary model and adjusted for sex, age, and daily gaming time to reduce confounding by gaming exposure. Model A adjusted only for sex and age, whereas Model C additionally included self-control, PHQ-2, and GAD-2. Complete coefficient tables for Models A and B are provided in Supplementary Table S2; Model C and the gamers-only sensitivity models are provided in Supplementary Table S3; residual and influence diagnostics are reported in Supplementary Table S4. Outcomes, escape-motivation scores, daily gaming time, self-control, PHQ-2, GAD-2, and the contextual variables were z-standardized; sex and age were entered using their original coding and units. We report standardized coefficients for the focal continuous variables, 95% confidence intervals, standard errors, exact *p* values, adjusted R-squared values, and variance inflation factors. Wald tests compared the two standardized escape-motivation coefficients within each model. Moderation analyses included escape motivation, self-control, and their interaction; because four interactions were examined, these results are exploratory. A sensitivity analysis was restricted to self-reported gamers (*n* = 270). Statistical significance was set at *p* < 0.05 (two-tailed). The analytic dataset contained no missing values on any study variable, and all analyses used complete cases (*n* = 358). All code was executed by the authors, and all coefficients, confidence intervals, and *p* values reported in the manuscript and supplementary tables were checked against the saved analysis output. For the contextual analysis, parental emotional warmth, parental autonomy support, parent–child relationship quality, and basic psychological need satisfaction were entered simultaneously in separate models for IGD symptoms and PSMU, with sex and age as covariates (Supplementary Table S5).

## 3 Results

### 3.1 Descriptive statistics and correlations

Table 1 presents descriptive statistics and bivariate correlations among the main variables. Game escape motivation and short-video escape motivation were strongly correlated (*r* = 0.75, *p* < 0.001), indicating that students who used one medium to escape tended to use the other similarly. However, their associations with the two outcomes diverged. Game escape motivation correlated more strongly with IGD symptoms (*r* = 0.40, *p* < 0.001) than with problematic social media use (PSMU; *r* = 0.31, *p* < 0.001), whereas short-video escape motivation correlated more strongly with PSMU (*r* = 0.37, *p* < 0.001) than with IGD symptoms (*r* = 0.28, *p* < 0.001). Self-control was negatively associated with both escape motivations and with both outcomes (*r*s = −0.35 to −0.41, all *p* < 0.001). A prespecified descriptive score threshold, 11.2% scored at or above the BSMAS threshold. This percentage describe the symptom-score distribution in this convenience sample and should not be interpreted as a diagnostic or prevalence estimate.

**Table 1:** Descriptive statistics and correlations (*n* = 358).

Variable	M	SD	1	2	3	4
1. Game escape	11.69	4.71	—			
2. Short-video escape	12.56	4.46	0.75***	—		
3. Self-control	38.58	6.44	−0.35***	−0.40***	—	
4. IGD symptoms	22.50	10.88	0.40***	0.28***	−0.39***	—
5. PSMU	15.45	5.76	0.31***	0.37***	−0.41***	0.51***

IGD = internet gaming disorder; PSMU = problematic social media use. ***p < 0.001.

### 3.2 Reliability, common method bias, and validation of the short-video escape measure

All scales showed good to excellent internal consistency (Cronbach’s *α*: game escape = 0.947; short-video escape = 0.949; self-control = 0.752; IGD = 0.973; PSMU = 0.943). The adapted IGD measure also showed a high mean inter-item correlation (0.770); corrected item–total correlations ranged from 0.811 to 0.914, and *α*-if-item-deleted values ranged from 0.969 to 0.972. Thus, all items contributed strongly to the total score, but the high inter-item correlations also indicate substantial content overlap; α = 0.973 should be interpreted as strong internal homogeneity rather than evidence of broad construct coverage. Harman’s single-factor test indicated that the first unrotated factor accounted for 26.63% of the variance. As an additional sensitivity check, a confirmatory single-factor model over all 42 substantive items fit very poorly (χ^2^(819) = 10301.1, CFI = 0.403, RMSEA = 0.180, SRMR = 0.173), far worse than a six-factor measurement model (χ^2^(804) = 3698.9, CFI = 0.818, RMSEA = 0.100, SRMR = 0.076). Common method variance was therefore assessed but cannot be excluded in an all-self-report design.

Because the short-video escape measure was newly adapted, we verified its structure. The four items were suitable for factor analysis (KMO = 0.82; Bartlett’s test, χ^2^ = 1459.0, *p* < 0.001), and a single principal component explained 86.7% of the variance (loadings 0.927–0.943). Structure was then tested with maximum-likelihood confirmatory factor analysis on the eight escape items. Because the short-video items were adapted in parallel from the game items with identical stems, the measurement model specified *a priori* included the four cross-scale parallel-item residual covariances (standardized residual correlations 0.25–0.39). This two-factor model showed good incremental and residual-based fit (χ^2^(15) = 78.0, CFI = 0.981, TLI = 0.964, SRMR = 0.021) and decisively outperformed the corresponding one-factor model (χ^2^(16) = 690.8, CFI = 0.794, SRMR = 0.077; Δχ^2^(1) = 612.8, *p* < 0.001), with a latent factor correlation of 0.78; the same comparison without the parallel residual covariances yielded χ^2^(19) = 190.4 versus χ^2^(20) = 716.3 (CFI = 0.948 vs. 0.787). Exact fit was rejected and RMSEA remained elevated in all models (0.108 for the *a priori* two-factor model). RMSEA may be less reliable in models with few degrees of freedom (26), while the residual correlations between parallel items indicate local dependence consistent with their closely matched wording. The structural interpretation therefore rests on the comparative test and the discriminant-validity indices below rather than on good absolute fit. Both measures showed strong convergent validity (game escape: AVE = 0.863, CR = 0.962; short-video escape: AVE = 0.867, CR = 0.963), and discriminant validity between the two was supported despite their correlation (HTMT = 0.79, below the 0.85 threshold). Mean inter-item correlations were, however, very high for both scales (0.82 for each), indicating that the near-ceiling alphas partly reflect closely parallel item wording; possible item redundancy is acknowledged as a limitation (Section 4.5). The two escape scales were therefore empirically distinguishable in this sample, although their high shared variance and parallel wording require cautious interpretation.

### 3.3 Medium-specific associations of escape motivation (H1)

**Figure 1:**
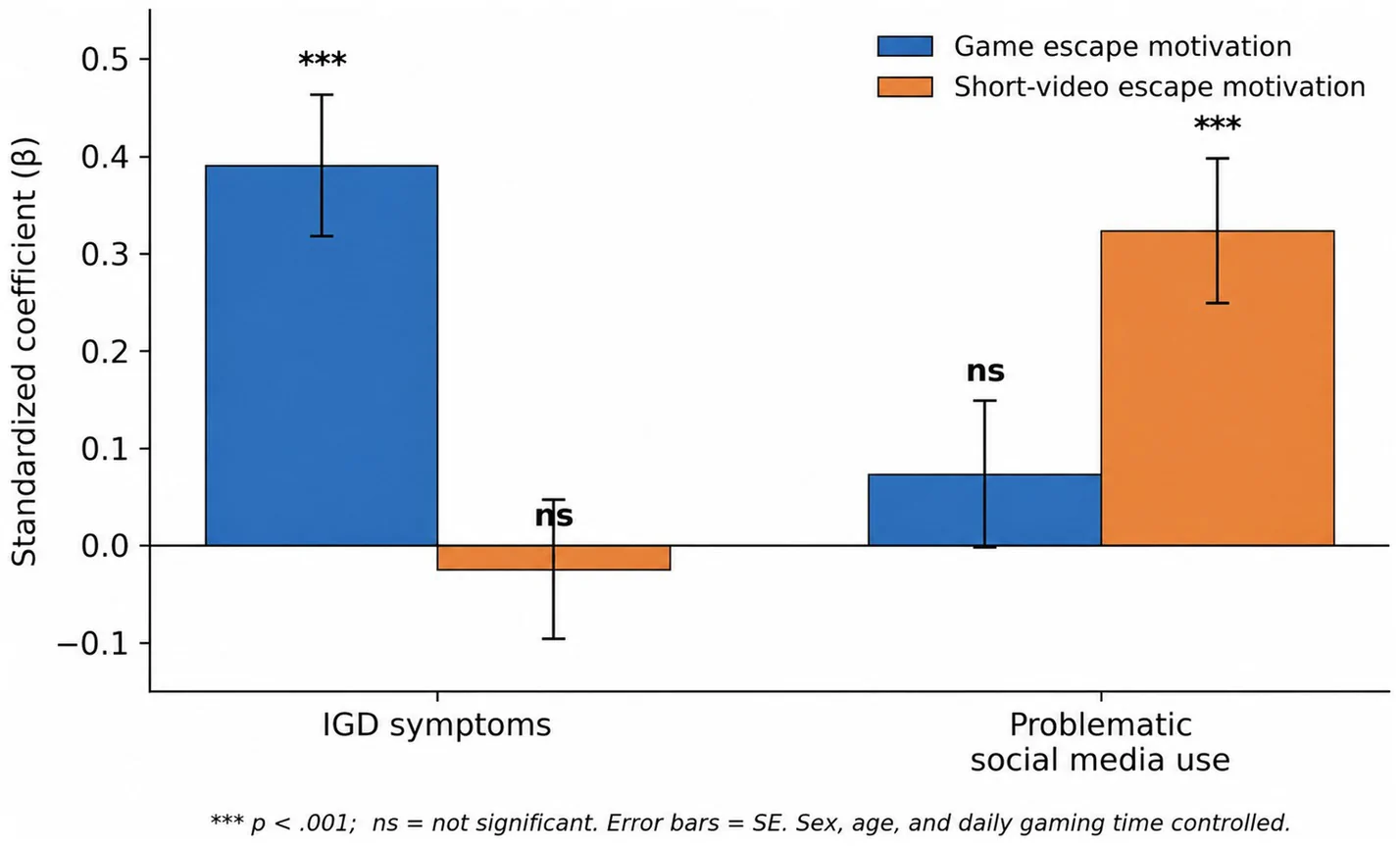
Primary Model B associations of game and short-video escape motivation with IGD symptoms and problematic social media use (*n* = 358). Standardized regression coefficients from models entering both escape motivations simultaneously and adjusting for sex, age, and daily gaming time. Error bars represent standard errors. *** *p* < 0.001; ns = not significant.

In the primary model adjusting for sex, age, and daily gaming time (Model B; Table 2), game escape motivation was associated with IGD symptoms (*β* = 0.340, 95% CI [0.198, 0.482], *p* < 0.001), whereas short-video escape motivation was not (*β* = 0.011, 95% CI [−0.128, 0.151], *p* = 0.872). The corresponding-medium coefficient was significantly larger than the noncorresponding coefficient (Δβ = 0.329, *F*(1, 352) = 6.02, *p* = 0.015). For PSMU, short-video escape motivation was associated with higher scores (*β* = 0.308, 95% CI [0.161, 0.456], *p* < 0.001), whereas game escape motivation was not (*β* = 0.095, 95% CI [−0.056, 0.245], *p* = 0.216). However, the corresponding-minus-noncorresponding coefficient difference was not significant [Δ*β* = 0.214, *F*(1, 352) = 2.27, *p* = 0.133]. Thus, formal evidence of differential medium specificity was present for IGD but not for PSMU. Because the PSMU outcome measures broad social-media problems rather than short-video-specific problematic use, the social-media-side finding is interpreted as preliminary (Figure 1).

**Table 2:** Primary Model B associations of escape motivation with IGD symptoms and PSMU (*n* = 358).

Predictor / test	IGD estimate	IGD *p*	PSMU estimate	PSMU *p*
Game escape motivation	0.340 [0.198, 0.482]	<0.001	0.095 [−0.056, 0.245]	0.216
Short-video escape motivation	0.011 [−0.128, 0.151]	0.872	0.308 [0.161, 0.456]	< 0.001
Δβ (Wald F)	0.329 (6.02)	0.015	0.214 (2.27)	0.133
Adjusted *R*^2^	0.239	—	0.146	—

Model B adjusts for sex, age, and daily gaming time. Estimates are standardized coefficients with 95% confidence intervals. For IGD, Δβ = βgame escape − βshort-video escape; for PSMU, Δβ = βshort-video escape − βgame escape. Positive values therefore indicate a larger hypothesized corresponding-medium coefficient. Δβ values were calculated from the unrounded coefficients; the component coefficients displayed in the table are rounded to three decimals. IGD = internet gaming disorder; PSMU = problematic social media use.

### 3.4 Self-control: negative associations and exploratory moderation (H2, H3)

Consistent with H2, self-control was strongly and negatively associated with both IGD symptoms (*β* = −0.29 to −0.31, *p* < 0.001) and PSMU (*β* = −0.30 to −0.33, *p* < 0.001) across models. Regarding moderation (H3), of the four escape × self-control interactions tested, only the interaction between game escape motivation and self-control in relation to IGD symptoms reached significance (*β* = −0.09, *p* = 0.016). This single interaction did not survive a Bonferroni correction for the four tests (*α* = 0.0125) and is therefore interpreted as exploratory. Descriptively, the association between game escape motivation and IGD symptoms was stronger at low self-control (−1 SD: slope = +0.36) than at high self-control (+1 SD: slope = +0.18). The remaining three interactions were not significant (*p*s = 0.10 to 0.36). Overall, self-control was a consistent correlate of lower problematic-use scores on both outcomes, whereas evidence of moderation was limited and preliminary (Table 3).

**Table 3:** Self-control as moderator of the escape–problem associations (*n* = 358).

Model	Escape *β*	Self-control *β*	Interaction *β*	Interaction *p*
Game escape × SC → IGD	0.27***	−0.29***	−0.09	0.016
Game escape × SC → PSMU	0.21***	−0.33***	0.07	0.104
Short-video escape × SC → IGD	0.15**	−0.31***	−0.03	0.358
Short-video escape × SC → PSMU	0.25***	−0.30***	0.05	0.156

Standardized coefficients; sex and age controlled. SC = self-control. **p < 0.01, ***p < 0.001.

### 3.5 Robustness and sensitivity analyses

Sensitivity analyses are reported in Supplementary Table S3. The minimally adjusted Model A produced the same qualitative pattern as the primary model. In the stringent sensitivity model (Model C), which additionally included self-control, PHQ-2, and GAD-2, the game escape–IGD association remained statistically significant (*β* = 0.37, *p* < 0.001), whereas the short-video escape–PSMU association was attenuated to non-significance (*β* = 0.13, *p* = 0.056). Because 24.6% of participants (*n* = 88) reported not playing games, the analyses were also repeated among self-reported gamers (*n* = 270). The game escape–IGD association was reproduced (*β* = 0.38, *p* < 0.001; short-video escape: *β* = 0.00, *p* = 0.980), whereas short-video escape motivation showed only marginal evidence of association with PSMU (*β* = 0.18, *p* = 0.055).

Across the primary and sensitivity models, the game escape–IGD association was robust after adjustment for daily gaming time. The inclusion of non-gamers was addressed separately by restricting the sensitivity analysis to self-reported gamers. The short-video escape–PSMU association was positive in the primary model but weaker under stringent adjustment and was not supported by a significant coefficient-difference test; it is therefore treated as preliminary.

### 3.6 Contextual analyses of family-related factors

In the contextual models, parental emotional warmth, parental autonomy support, parent–child relationship quality, and basic psychological need satisfaction were entered simultaneously with sex and age as covariates. None of the four focal variables was independently associated with IGD symptoms (|*β*| ≤ 0.064, *p*s ≥ 0.507) or PSMU (|*β*| ≤ 0.146, *p*s ≥ 0.216; Supplementary Table S5).

## 4 Discussion

This study compared the differential associations of game escape motivation and short-video escape motivation with internet gaming disorder (IGD) symptoms and problematic social media use (PSMU), and further examined the main and moderating roles of self-control and the contribution of current family-related and contextual factors in an emerging-adult sample. After both escape motives and daily gaming time were considered, game escape motivation remained associated with IGD symptoms, short-video escape motivation was not, and the difference between the two coefficients was significant. Short-video escape motivation was associated with PSMU, whereas game escape motivation was not; however, the coefficient difference was non-significant and the short-video association weakened under stringent adjustment. Self-control was consistently negatively associated with both outcomes and showed preliminary moderation of the game escape-IGD association. None of the four contextual variables showed an independent direct association when entered simultaneously. Taken together, the findings support partial and asymmetric medium specificity of escape motivation and suggest that self-regulatory capacity and developmental context jointly shape problematic technology use.

### 4.1 Partial and asymmetric medium specificity of escape motivation

Escape motivation has long been regarded as one of the most important motivational correlates of gaming disorder. Two meta-analyses and a systematic review found that escape or escapism generally showed stronger associations with gaming-disorder symptoms than achievement, social, or entertainment motives (27–29). A three-level meta-analysis of problematic short-video use likewise identified escapism as the motivation with the strongest pooled association (30). These studies separately support the correspondence between game escape and gaming problems and between short-video escape and short-video problems. However, because previous studies have usually examined motives and problematic behavior within a single medium, they do not directly distinguish a general tendency to escape from medium-specific escape motivation.

The present study entered game escape motivation and short-video escape motivation simultaneously, allowing their unique associations to be examined after their shared variance was controlled. The IGD-side finding was clear: game escape motivation remained associated with IGD symptoms after adjustment for short-video escape motivation and daily gaming time, and its coefficient was significantly larger than that of short-video escape motivation. Thus, game escape motivation appears to contain a gaming-specific component that is not reducible to a general escape tendency or greater gaming exposure. Related evidence has also shown that escapism can help distinguish gaming-disorder risk from playtime alone among highly engaged gamers (31).

The PSMU-side findings were directionally consistent but less conclusive. Short-video escape motivation was associated with PSMU in the primary model, whereas game escape motivation was not. This pattern accords with longitudinal evidence that coping-motivated social-media use predicts subsequent increases in PSMU and with recent findings linking coping motives to PSMU among Chinese college students (32, 33). Nevertheless, the difference between the two escape-motivation coefficients did not reach significance, and the short-video association weakened to non-significance after additional adjustment for self-control, depression, and anxiety. The medium-specificity hypothesis was therefore clearly supported on the IGD side and received directional support on the PSMU side.

The asymmetric strength of the two findings may partly reflect differences in measurement correspondence. Game escape motivation was paired with an IGD-specific outcome, whereas short-video escape motivation was paired with the BSMAS, which measures broad social-media problems rather than problematic short-video use specifically. PSMU scores may therefore capture difficulties arising from social-networking sites, messaging services, and other platforms in addition to short-video use. This construct mismatch may have attenuated the coefficient difference and reduced the stability of the short-video association under stringent adjustment.

At the same time, the two escape-motivation measures were strongly correlated, suggesting that individuals may also possess a general tendency to use digital activities for escape. The findings are therefore consistent with a layered structure: shared emotional distress, need frustration, or avoidance tendencies may promote compensatory engagement across digital activities, while the psychological affordances, reinforcement schedules, and habits associated with particular media concentrate part of that risk within a specific activity. This interpretation is consistent with the compensatory internet-use framework and the I-PACE model, both of which allow common vulnerabilities and behavior-specific mechanisms to operate together (7, 34). The present results support partial and asymmetric medium specificity: game escape motivation showed a comparatively clear IGD-specific component, whereas specificity on the short-video side requires further testing with a short-video-specific problematic-use measure.

### 4.2 Cross-domain effects and conditional moderation of self-control

Self-control was negatively associated with both IGD symptoms and PSMU, supporting its role as a cross-domain self-regulatory factor. A meta-analysis found a consistent association between lower self-control and problematic internet use among students (12). Broader syntheses of gaming-disorder risk factors and longitudinally modifiable factors have likewise identified self-regulatory deficits as relatively consistent individual-level correlates of problematic gaming (35, 36). In the present study, the main associations of self-control were similar across the two outcomes, suggesting that self-control may operate through general processes such as impulse inhibition, goal maintenance, and delay of gratification rather than being restricted to a particular digital medium.

**Figure 2:**
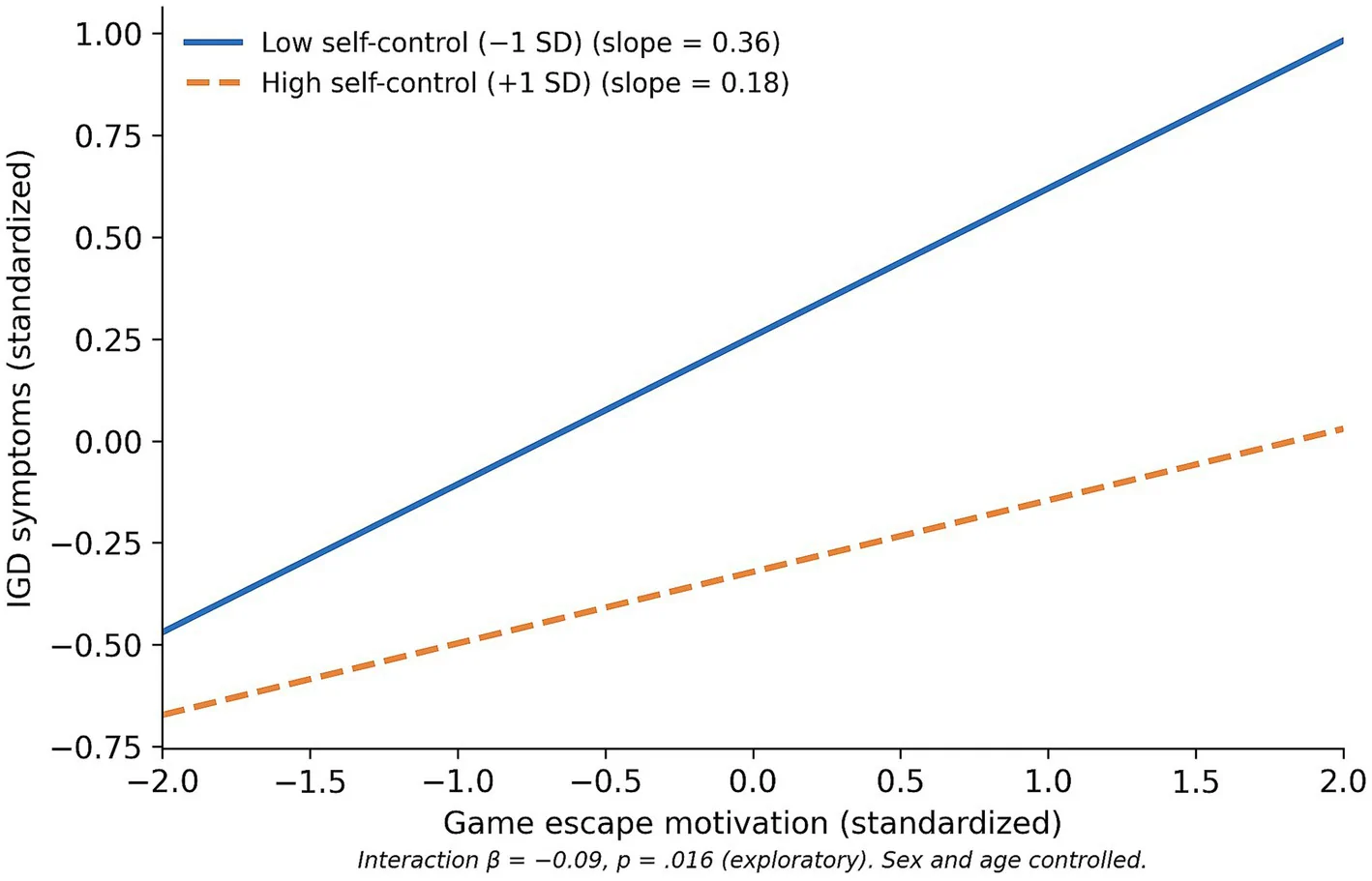
Simple slopes for the association between game escape motivation and IGD symptoms at low (−1 SD) and high (+1 SD) levels of self-control. The interaction was exploratory (*β* = −0.09, *p* = 0.016) and did not survive correction for multiple testing.

The interaction between game escape motivation and self-control further indicated that the association between game escape motivation and IGD symptoms was stronger at lower levels of self-control and weaker at higher levels. As illustrated in Figure 2, self-control may condition whether the desire to escape through gaming is expressed as persistent, poorly controlled gaming that produces adverse consequences. This interpretation is consistent with the I-PACE model, in which inhibitory control influences the translation of affective and cognitive responses into problematic behavior (34).

The interaction emerged only for the corresponding game escape-IGD pathway and was not observed across all motive-outcome combinations. One possible explanation is that moderation by self-control is more detectable when the motive and problematic behavior are closely matched. The main association and coefficient difference were most stable for game escape and IGD, whereas the noncorresponding paths were weaker and the PSMU-side test was affected by imperfect measurement correspondence. The interaction was small, was the only conventionally significant result among four interaction tests, and did not survive Bonferroni correction. It therefore provides preliminary evidence of conditional moderation rather than proof of a stable causal buffering mechanism. Preregistered studies combining behavioral inhibition tasks, digital-use logs, and longitudinal data are needed to determine whether higher self-control prospectively weakens the transition from escape motivation to IGD.

### 4.3 Developmental reorganization of family influence in emerging adulthood

Parental emotional warmth, parental autonomy support, parent–child relationship quality, and basic psychological need satisfaction showed no independent direct associations with IGD or PSMU in the simultaneous contextual models. The null findings for the family-related variables differ from a substantial adolescent literature. Systematic reviews and meta-analyses generally associate warmer, more supportive, and better-functioning family environments with lower problematic internet use and IGD among adolescents (13, 14). Prospective studies have also identified potentially protective associations of parental warmth, monitoring, and support, and research with Chinese adolescents has linked parental emotional warmth to lower IGD through self-regulatory capacities such as time management (37–39).

This difference may reflect a developmental reorganization of family influence. Emerging adulthood involves greater autonomy in residence, behavioral decisions, and interpersonal relationships, while peers, current stressors, and individual self-regulation become increasingly proximal to everyday digital behavior (15). Consistent with this account, a three-wave study of first-year undergraduates found that mother and father attachment did not predict subsequent IGD, whereas peer attachment did (40). In the present study, escape motivation and self-control also showed stronger direct associations with problematic use than the unique contribution of the current family-related variables, which is compatible with individual and peer-level processes becoming more proximal during the university years.

Family influence, however, does not necessarily disappear in university populations. Recent longitudinal research with Chinese university students found that family functioning predicted later PSMU both directly and indirectly through loneliness, and that positive parenting moderated a pathway from negative life events to PSMU through fear of missing out (41, 42). A large university study also linked family functioning to social-media addiction through depressive symptoms, with peer support modifying part of this pathway (43). These findings suggest that family effects during emerging adulthood may be more readily observed as total, mediated, or conditional effects than as the independent direct effect of a single current family variable.

Differences in modeling and measurement may further explain the contrasting findings. In the present study, several related contextual variables were entered simultaneously, so each coefficient represented a unique association after the remaining variables were controlled; studies based on a single family-functioning index estimate a broader overall association. Family functioning, parental warmth, autonomy support, and positive parenting also capture different processes and are not interchangeable. Family effects may be concentrated among students who continue to live with or depend financially on parents, experience marked loneliness or stress, or have particularly poor family relationships, and such subgroup or conditional effects may be obscured in an average main-effect model. The form of parental involvement is also important, because controlling parental mediation may strengthen rather than reduce the association between escape and gaming-disorder risk (44).

Taken together, the evidence suggests that family influence in emerging adulthood may shift from a broadly direct and contemporaneous effect toward more distal, indirect, and conditional forms. Earlier family experiences may continue to shape digital behavior through self-control, emotion regulation, and relational patterns, whereas the direct influence of the current family environment may depend on residential status, continuing dependence, stress exposure, and the quality of family interaction.

### 4.4 Implications for research and practice

The findings suggest that assessment of problematic technology use should consider not only the amount of time spent online but also the medium used to regulate negative affect and whether escape motivation is concentrated within a particular activity. The robust gaming-side result supports the joint use of gaming-exposure indicators and game-specific motive measures in IGD research. The short-video finding likewise indicates potential value in matching motive assessment to the medium, although a short-video-specific problematic-use scale is needed before the approach can be evaluated for risk identification.

Self-control may represent a cross-medium factor for risk identification, while the combination of strong game escape motivation and low self-control may identify a more concentrated IGD risk profile. Future intervention studies could compare general self-regulation training with context-specific approaches. In addition to strengthening impulse control, such interventions could help students recognize escape-related gaming urges following academic stress, interpersonal setbacks, or negative affect and develop alternative emotion-regulation responses.

The family-related findings also suggest that intervention strategies should be calibrated to developmental stage. For adolescents, improving parental warmth, support, and appropriate monitoring remains well supported. During the university years, family involvement may be most useful when tailored to students’ residential status, financial dependence, family conflict, and communication quality while respecting emerging autonomy. Self-regulation, peer support, loneliness, and current stress may be more proximal targets for students who live away from home and function relatively independently, whereas autonomy-supportive family communication may remain valuable when family relationships continue to play an active role.

### 4.5 Limitations

The findings should be interpreted in light of several limitations. The cross-sectional design cannot establish the temporal ordering of escape motivation, self-control, family-related factors, and problematic technology use, and reverse or reciprocal relationships remain possible; for example, persistent problematic use may intensify the wish to escape, undermine self-control, or alter perceptions of family relationships. Participants were recruited from a single university in Shandong Province and the sample included a larger proportion of women, so replication is needed across regions, institutional types, residential arrangements, and levels of continuing family dependence. All variables were assessed through self-report at one time point. Although a single-factor model did not adequately account for the observed covariance, common-method variance, recall bias, and social-desirability effects cannot be excluded, and future studies should incorporate digital-use logs, behavioral inhibition tasks, and parent or peer reports. Short-video escape motivation was paired with the BSMAS, which measures broad PSMU rather than problematic short-video use specifically; this incomplete predictor-outcome match may have weakened the short-video coefficient difference and may partly explain why the evidence was less stable than on the gaming side. Although daily gaming time was controlled and the game escape-IGD association was reproduced among self-reported gamers, motivation and behavioral exposure cannot be fully separated without objective logs of frequency, duration, and situational use. The contextual model estimated the independent direct association of each variable after the other family-related and need-satisfaction variables were controlled; the results therefore do not exclude a combined family effect, indirect effects through loneliness, self-control, or emotional distress, or effects confined to particular residential, dependence, or stress subgroups. Several features of the measurement and analytic design also warrant consideration. The source GADIS-A was originally developed for adolescents, although the Chinese version has psychometric support in emerging adults. The two escape-motivation scales used highly parallel wording, and their very high internal consistencies may partly reflect overlapping item content. In addition, the short-video escape measure was reviewed by bilingual researchers but was not subjected to formal expert content-validity ratings, pilot testing, or cognitive interviewing before administration. The self-control interaction was small, was observed in only one of four tests, and did not survive correction for multiple comparisons, so independent preregistered replication is required. Religiosity and religious belonging were also not assessed. Existing evidence specifically suggests that religiosity may be associated with lower gaming-disorder symptoms and better mental-health outcomes (45). The potential roles of religiosity and religious belonging warrant examination in cultural and educational contexts in which such assessment is appropriate.

## 5 Conclusion

Among Chinese university students, escape motivation showed partial and asymmetric medium specificity. Game escape motivation remained associated with IGD symptoms after short-video escape motivation and daily gaming time were controlled, and its association was significantly stronger than that of short-video escape motivation, providing clear support for the gaming side of the medium-specificity hypothesis. The association between short-video escape motivation and PSMU was directionally consistent with the hypothesis, but the non-significant coefficient difference, attenuation under stringent adjustment, and use of a broad rather than short-video-specific outcome leave the social-media side in need of further confirmation.

Self-control was negatively associated with both IGD symptoms and PSMU and showed preliminary moderation of the game escape-IGD association, suggesting that general self-regulatory capacity and medium-specific escape motivation may jointly contribute to problematic use. Current family-related variables showed no independent direct associations. When considered alongside evidence from adolescent and university samples, this pattern is consistent with a developmental reorganization of family influence: direct contemporaneous effects may weaken, while effects transmitted through self-control, emotional distress, loneliness, continuing dependence, stressful circumstances, and the quality of family interaction may persist.

Overall, problematic technology use may reflect the joint contribution of a cross-medium vulnerability to escape, the escape functions afforded by particular media, individual self-regulatory capacity, and the surrounding developmental and family context. Longitudinal studies using fully medium-matched measures, multi-source behavioral indicators, and more representative samples are needed to clarify the temporal relations among these factors and their value for risk identification and intervention.

## Data Availability

The datasets presented in this study can be found in online repositories. The names of the repository/repositories and accession number(s) can be found in the article/Supplementary material.

## Footnotes

1
https://doi.org/10.17605/OSF.IO/YW3TB

## References

[1] World Health Organization. International Classification of Diseases for Mortality and Morbidity Statistics (11th Revision). Geneva: World Health Organization (2019).

[2] American Psychiatric Association. Diagnostic and Statistical Manual of Mental Disorders. 5th ed. Washington, DC: American Psychiatric Publishing (2013).

[3] Paulus FW Ohmann S von Gontard A Popow C. Internet gaming disorder in children and adolescents: a systematic review. Dev Med Child Neurol. (2018) 60:645–59. doi: 10.1111/dmcn.13754 29633243

[4] Andreassen CS Billieux J Griffiths MD Kuss DJ Demetrovics Z Mazzoni E . The relationship between addictive use of social media and video games and symptoms of psychiatric disorders: a large-scale cross-sectional study. Psychol Addict Behav. (2016) 30:252–62. doi: 10.1037/adb0000160, 26999354

[5] Demetrovics Z Urbán R Nagygyörgy K Farkas J Zilahy D Mervó B . Why do you play? The development of the motives for online gaming questionnaire (MOGQ). Behav Res Methods. (2011) 43:814–25. doi: 10.3758/s13428-011-0091-y, 21487899

[6] Laconi S Pirès S Chabrol H. Internet gaming disorder, motives, game genres and psychopathology. Comput Human Behav. (2017) 75:652–9. doi: 10.1016/j.chb.2017.06.012

[7] Kardefelt-Winther D. A conceptual and methodological critique of internet addiction research: towards a model of compensatory internet use. Comput Human Behav. (2014) 31:351–4. doi: 10.1016/j.chb.2013.10.059

[8] Montag C Yang H Elhai JD. On the psychology of TikTok use: a first glimpse from empirical findings. Front Public Health. (2021) 9:641673. doi: 10.3389/fpubh.2021.641673, 33816425 PMC8010681

[9] Su C Zhou H Gong L Teng B Geng F Hu Y. Viewing personalized video clips recommended by TikTok activates default mode network and ventral tegmental area. NeuroImage. (2021) 237:118136. doi: 10.1016/j.neuroimage.2021.118136, 33951514

[10] China Internet Network Information Center (CNNIC) (2025) The 55th statistical report on China’s internet development. Available online at: https://www.cnnic.com.cn/IDR/ReportDownloads/202505/P020250514564119130448.pdf (Accessed July 24, 2026).

[11] Tangney JP Baumeister RF Boone AL. High self-control predicts good adjustment, less pathology, better grades, and interpersonal success. J Pers. (2004) 72:271–324. doi: 10.1111/j.0022-3506.2004.00263.x, 15016066

[12] Li S Ren P Chiu MM Wang C Lei H. The relationship between self-control and internet addiction among students: a meta-analysis. Front Psychol. (2021) 12:735755. doi: 10.3389/fpsyg.2021.735755, 34899477 PMC8653951

[13] Schneider LA King DL Delfabbro PH. Family factors in adolescent problematic internet gaming: a systematic review. J Behav Addict. (2017) 6:321–33. doi: 10.1556/2006.6.2017.035, 28762279 PMC5700711

[14] Lukavská K Hrabec O Lukavský J Demetrovics Z Király O. The associations of adolescent problematic internet use with parenting: a meta-analysis. Addict Behav. (2022) 135:107423. doi: 10.1016/j.addbeh.2022.107423, 35933287

[15] Arnett JJ. Emerging adulthood: a theory of development from the late teens through the twenties. Am Psychol. (2000) 55:469–80. doi: 10.1037/0003-066X.55.5.469, 10842426

[16] Wu AMS Lai MHC Yu S Lau JTF Lei MW. Motives for online gaming questionnaire: its psychometric properties and correlation with internet gaming disorder symptoms among Chinese people. J Behav Addict. (2017) 6:11–20. doi: 10.1556/2006.6.2017.007, 28264590 PMC5572999

[17] Tan S Guo Y. Revision of the self-control scale for Chinese college students [大学生自我控制量表的修订]. Chin J Clin Psychol. (2008) 16:468–70. doi: 10.16128/j.cnki.1005-3611.2008.05.022

[18] Paschke K Austermann MI Thomasius R. Assessing ICD-11 gaming disorder in adolescent gamers: development and validation of the gaming disorder scale for adolescents (GADIS-A). J Clin Med. (2020) 9:993. doi: 10.3390/jcm9040993, 32252305 PMC7230491

[19] Chen IH Chang YL Yang YN Yeh YC Ahorsu DK Adjorlolo S . Psychometric properties and development of the Chinese versions of gaming disorder test (GDT) and gaming disorder scale for adolescents (GADIS-A). Asian J Psychiatr. (2023) 86:103638. doi: 10.1016/j.ajp.2023.103638, 37285663

[20] Bányai F Zsila Á Király O Maraz A Elekes Z Griffiths MD . Problematic social media use: results from a large-scale nationally representative adolescent sample. PLoS One. (2017) 12:e0169839. doi: 10.1371/journal.pone.0169839, 28068404 PMC5222338

[21] Luo T Qin L Cheng L Wang S Zhu Z Xu J . Determination the cut-off point for the Bergen social media addiction (BSMAS): diagnostic contribution of the six criteria of the components model of addiction for social media disorder. J Behav Addict. (2021) 10:281–90. doi: 10.1556/2006.2021.00025, 34010148 PMC8996805

[22] Jiang J Lu Z Jiang B Xu Y. Preliminary revision of the Chinese version of the short-form Egna Minnen av Barndoms Uppfostran [简式父母教养方式问卷中文版的初步修订]. Psychol Dev Educ. (2010) 26:94–9. doi: 10.16187/j.cnki.issn1001-4918.2010.01.017

[23] Chen B Vansteenkiste M Beyers W Boone L Deci EL Van der Kaap-Deeder J . Basic psychological need satisfaction, need frustration, and need strength across four cultures. Motiv Emot. (2015) 39:216–36. doi: 10.1007/s11031-014-9450-1

[24] Kroenke K Spitzer RL Williams JB. The patient health Questionnaire-2: validity of a two-item depression screener. Med Care. (2003) 41:1284–92. doi: 10.1097/01.MLR.0000093487.78664.3C, 14583691

[25] Kroenke K Spitzer RL Williams JB Monahan PO Löwe B. Anxiety disorders in primary care: prevalence, impairment, comorbidity, and detection. Ann Intern Med. (2007) 146:317–25. doi: 10.7326/0003-4819-146-5-200703060-00004, 17339617

[26] Kenny DA Kaniskan B McCoach DB. The performance of RMSEA in models with small degrees of freedom. Sociol Methods Res. (2015) 44:486–507. doi: 10.1177/0049124114543236

[27] Wang HY Cheng C. The associations between gaming motivation and internet gaming disorder: systematic review and meta-analysis. JMIR Ment Health. (2022) 9:e23700. doi: 10.2196/23700, 35175204 PMC8895288

[28] Bäcklund C Elbe P Gavelin HM Sörman DE Ljungberg JK. Gaming motivations and gaming disorder symptoms: a systematic review and meta-analysis. J Behav Addict. (2022) 11:667–88. doi: 10.1556/2006.2022.00053, 36094861 PMC9872536

[29] Melodia F Canale N Griffiths MD. The role of avoidance coping and escape motives in problematic online gaming: a systematic literature review. Int J Ment Health Addict. (2022) 20:996–1022. doi: 10.1007/s11469-020-00422-w

[30] Chen Y Zhang W Zhong N Zhao M. Motivations behind problematic short video use: a three-level meta-analysis. Telemat Inform. (2024) 95:102196. doi: 10.1016/j.tele.2024.102196

[31] Kiszka P Strojny A Strojny P. High immersion/escapism motivation makes gaming disorder risk less dependent of playtime among highly engaged male gamers. Front Psych. (2024) 15:1443091. doi: 10.3389/fpsyt.2024.1443091, 39386891 PMC11461240

[32] Moretta T Buodo G Santucci VG Chen S Potenza MN. Problematic social media use is statistically predicted by using social media for coping motives and by positive reinforcement processes in individuals with high COVID-19-related stress levels. J Psychiatr Res. (2023) 158:104–13. doi: 10.1016/j.jpsychires.2022.12.036, 36580866

[33] Li M Hao Y Wang Y Morris R 3rd Wang X Liu T . Prevalence, motivation, psychological distress, and gender differences of problematic social media use among Chinese college students. J Psychiatr Res. (2025) 191:306–12. doi: 10.1016/j.jpsychires.2025.09.056 41037974

[34] Brand M Wegmann E Stark R Müller A Wölfling K Robbins TW . The interaction of person-affect-cognition-execution (I-PACE) model for addictive behaviors: update, generalization to addictive behaviors beyond internet-use disorders, and specification of the process character of addictive behaviors. Neurosci Biobehav Rev. (2019) 104:1–10. doi: 10.1016/j.neubiorev.2019.06.032, 31247240

[35] Ropovik I Martončik M Babinčák P Baník G Vargová L Adamkovič M. Risk and protective factors for (internet) gaming disorder: a meta-analysis of pre-COVID studies. Addict Behav. (2023) 139:107590. doi: 10.1016/j.addbeh.2022.107590, 36571943

[36] Zhuang X Zhang Y Tang X Ng TK Lin J Yang X. Longitudinal modifiable risk and protective factors of internet gaming disorder: a systematic review and meta-analysis. J Behav Addict. (2023) 12:375–92. doi: 10.1556/2006.2023.00017, 37224007 PMC10316169

[37] Lukavská K Vacek J Gabrhelík R. The effects of parental control and warmth on problematic internet use in adolescents: a prospective cohort study. J Behav Addict. (2020) 9:664–75. doi: 10.1556/2006.2020.00068, 32976113 PMC8943671

[38] She R Zhang Y Yang X. Parental factors associated with internet gaming disorder among first-year high school students: longitudinal study. JMIR Serious Games. (2022) 10:e33806. doi: 10.2196/33806, 36346660 PMC9682450

[39] Chen IH Lee ZH Dong XY Gamble JH Feng HW. The influence of parenting style and time management tendency on internet gaming disorder among adolescents. Int J Environ Res Public Health. (2020) 17:9120. doi: 10.3390/ijerph17239120, 33291336 PMC7730530

[40] Teng Z Griffiths MD Nie Q Xiang G Guo C. Parent-adolescent attachment and peer attachment associated with internet gaming disorder: a longitudinal study of first-year undergraduate students. J Behav Addict. (2020) 9:116–28. doi: 10.1556/2006.2020.00011, 32359235 PMC8935186

[41] Lu H Ren Z Zhen P Su Z Wu Y. The longitudinal association between family functioning and problematic social media use among Chinese university students: mediation via loneliness and a subgroup analysis by sex. Addict Behav. (2025) 166:108337. doi: 10.1016/j.addbeh.2025.108337, 40121924

[42] Yuan XQ Dou K Li YY. The longitudinal association between negative life events and problematic social media use among Chinese college students: the mediating role of FoMO and the moderating role of positive parenting. Stress Health. (2024) 40:e3505. doi: 10.1002/smi.3505, 39572931

[43] Qi Y Zhao M Geng T Tu Z Lu Q Li R . The relationship between family functioning and social media addiction among university students: a moderated mediation model of depressive symptoms and peer support. BMC Psychol. (2024) 12:341. doi: 10.1186/s40359-024-01818-2, 38858753 PMC11165749

[44] Görgülü Z Özer A. Conditional role of parental controlling mediation on the relationship between escape, daily game time, and gaming disorder. Curr Psychol. (2024) 43:3821–9. doi: 10.1007/s12144-023-04557-6, 37359689 PMC10091322

[45] Chakhunashvili K Chakhunashvili DG Patil RR Mchedlishvili E Romem-Porat SL Reznik A . Gaming, religiosity and mental health among Georgian students. Discov Ment Health. (2025) 5:157. doi: 10.1007/s44192-025-00297-0, 41123755 PMC12545947

