# Supplementary material for: Medium-specific escape motivation in internet gaming disorder and problematic social media use among Chinese university students: the role of self-control
Source: Front Public Health. 2026 Aug 26;14:1896731. doi: 10.3389/fpubh.2026.1896731 (PMC13553412; doi:10.3389/fpubh.2026.1896731)
Supplement: Supplementary file 1 [file Table_2.DOCX]

# Supplementary Material

**Medium-Specific Escape Motivation in Internet Gaming Disorder and Problematic Social Media Use Among Chinese University Students: The Role of Self-Control**

## Supplementary Table S1. Item wording and adaptation procedure for the escape-motivation measures

Adaptation procedure: the four short-video escape items were created from the four game escape items (MOGQ escape subscale; Chinese version Wu et al., 2017) by substituting the activity context (“playing games” → “watching short videos”) while keeping the remaining item content and response format unchanged. Two bilingual members of the research team independently reviewed the modified wording for semantic equivalence and contextual appropriateness, and differences were resolved through discussion. No formal expert-panel content-validity rating, pilot study, or cognitive interviewing was conducted before data collection. Response scale: 1 = never, 2 = occasionally, 3 = sometimes, 4 = often, 5 = always. Stems: “I play online games in order to …” (game items) / “I watch short videos in order to …” (short-video items).

| **#** | **Game escape item (Chinese, as administered)** | **Short-video escape item (Chinese, as administered)** | **English gloss** |
| --- | --- | --- | --- |
| 1 | 忘记现实中的烦恼和压力 | 忘记现实中的烦恼和压力 | … to forget real-life worries and stress |
| 2 | 逃避现实生活中我不喜欢面对的事情 | 逃避现实生活中我不喜欢面对的事情 | … to avoid things in real life I do not want to face |
| 3 | 当我感到难过或情绪低落时，让自己感觉好一些 | 当我感到难过或情绪低落时，让自己感觉好一些 | … to feel better when I feel sad or down |
| 4 | 暂时摆脱学习或人际关系中的困扰 | 暂时摆脱学习或人际关系中的困扰 | … to temporarily get away from troubles in schoolwork or interpersonal relationships |

## Supplementary Table S2. Complete regression models A and B (n = 358)

IGD symptoms — Model A (adj. R² = .206, F(4, 353) = 24.2)

| **Predictor** | **Estimate** | **SE** | **t** | **p** | **95% CI** | **VIF** |
| --- | --- | --- | --- | --- | --- | --- |
| Game escape | .391 | .073 | 5.38 | < .001 | [.248, .533] | 2.37 |
| Short-video escape | −.025 | .072 | −0.34 | .731 | [−.166, .116] | 2.31 |
| Sex | −.483 | .101 | −4.80 | < .001 | [−.680, −.285] | 1.04 |
| Age | −.054 | .033 | −1.63 | .103 | [−.119, .011] | 1.02 |

IGD symptoms — Model B (primary; adj. R² = .239, F(5, 352) = 23.4)

| **Predictor** | **Estimate** | **SE** | **t** | **p** | **95% CI** | **VIF** |
| --- | --- | --- | --- | --- | --- | --- |
| Game escape | .340 | .072 | 4.71 | < .001 | [.198, .482] | 2.45 |
| Short-video escape | .011 | .071 | 0.16 | .872 | [−.128, .151] | 2.35 |
| Daily gaming time | .204 | .051 | 4.03 | < .001 | [.104, .303] | 1.20 |
| Sex | −.337 | .105 | −3.21 | .001 | [−.543, −.131] | 1.18 |
| Age | −.035 | .033 | −1.07 | .284 | [−.099, .029] | 1.05 |

PSMU — Model A (adj. R² = .142, F(4, 353) = 15.8)

| **Predictor** | **Estimate** | **SE** | **t** | **p** | **95% CI** | **VIF** |
| --- | --- | --- | --- | --- | --- | --- |
| Game escape | .073 | .075 | 0.97 | .331 | [−.075, .222] | 2.37 |
| Short-video escape | .323 | .075 | 4.34 | < .001 | [.177, .470] | 2.31 |
| Sex | −.034 | .105 | −0.33 | .743 | [−.240, .171] | 1.04 |
| Age | −.076 | .034 | −2.21 | .028 | [−.144, −.008] | 1.02 |

PSMU — Model B (primary; adj. R² = .146, F(5, 352) = 13.2)

| **Predictor** | **Estimate** | **SE** | **t** | **p** | **95% CI** | **VIF** |
| --- | --- | --- | --- | --- | --- | --- |
| Game escape | .095 | .076 | 1.24 | .216 | [−.056, .245] | 2.45 |
| Short-video escape | .308 | .075 | 4.11 | < .001 | [.161, .456] | 2.35 |
| Daily gaming time | −.086 | .054 | −1.60 | .111 | [−.191, .020] | 1.20 |
| Sex | −.095 | .111 | −0.86 | .391 | [−.314, .123] | 1.18 |
| Age | −.084 | .035 | −2.42 | .016 | [−.152, −.016] | 1.05 |

Note. Outcomes, escape-motivation scores, and daily gaming time were z-standardized; sex and age were entered using their original coding and units. Positive Δβ values indicate a larger hypothesized corresponding-medium coefficient (IGD: βgame escape − βshort-video escape; PSMU: βshort-video escape − βgame escape). Model A Wald tests: IGD Δβ = .415, F(1, 353) = 9.46, p = .002; PSMU Δβ = .250, F(1, 353) = 3.17, p = .076. Model B Wald tests: IGD Δβ = .329, F(1, 352) = 6.02, p = .015; PSMU Δβ = .214, F(1, 352) = 2.27, p = .133.

## Supplementary Table S3. Complete fully adjusted models (Model C) and gamers-only sensitivity models

IGD symptoms — Model C (adj. R² = .367, F(8, 349) = 26.9)

| **Predictor** | **Estimate** | **SE** | **t** | **p** | **95% CI** | **VIF** |
| --- | --- | --- | --- | --- | --- | --- |
| Game escape | .370 | .066 | 5.58 | < .001 | [.239, .500] | 2.47 |
| Short-video escape | −.135 | .067 | −2.00 | .046 | [−.267, −.002] | 2.56 |
| Daily gaming time | .151 | .047 | 3.22 | .001 | [.059, .243] | 1.24 |
| Self-control | −.154 | .050 | −3.11 | .002 | [−.251, −.057] | 1.38 |
| PHQ-2 | .230 | .086 | 2.67 | .008 | [.061, .400] | 4.19 |
| GAD-2 | .085 | .085 | 1.00 | .318 | [−.082, .251] | 4.04 |
| Sex | −.267 | .096 | −2.78 | .006 | [−.456, −.078] | 1.19 |
| Age | −.017 | .030 | −0.58 | .563 | [−.076, .042] | 1.06 |

PSMU — Model C (adj. R² = .340, F(8, 349) = 23.9)

| **Predictor** | **Estimate** | **SE** | **t** | **p** | **95% CI** | **VIF** |
| --- | --- | --- | --- | --- | --- | --- |
| Game escape | .126 | .068 | 1.86 | .064 | [−.007, .259] | 2.47 |
| Short-video escape | .132 | .069 | 1.92 | .056 | [−.003, .267] | 2.56 |
| Daily gaming time | −.149 | .048 | −3.12 | .002 | [−.243, −.055] | 1.24 |
| Self-control | −.211 | .051 | −4.17 | < .001 | [−.311, −.111] | 1.38 |
| PHQ-2 | .189 | .088 | 2.14 | .033 | [.016, .362] | 4.19 |
| GAD-2 | .181 | .086 | 2.10 | .037 | [.011, .351] | 4.04 |
| Sex | −.010 | .098 | −0.10 | .919 | [−.203, .183] | 1.19 |
| Age | −.065 | .031 | −2.11 | .035 | [−.125, −.004] | 1.06 |

Gamers-only sensitivity models (Model A specification; n = 270)

| Outcome | Game escape β (p) | Short-video escape β (p) |
| --- | --- | --- |
| IGD symptoms | .378 (< .001) | −.002 (.980) |
| PSMU | .138 (.132) | .175 (.055) |

Note. In Model C, outcomes, escape-motivation scores, daily gaming time, self-control, PHQ-2, and GAD-2 were z-standardized; sex and age were entered using their original coding and units. In Model C, the PHQ-2 and GAD-2 VIFs (≈ 4.2 and 4.0) reflect the expected overlap of the two affect screeners; all other VIFs ≤ 2.6. Model C Wald tests were: IGD Δβ = .504, F(1, 349) = 16.55, p < .001; PSMU Δβ = .006, F(1, 349) = 0.00, p = .963. The negative short-video coefficient for IGD in Model C is a conditional (suppression-type) estimate arising under simultaneous control of game escape, affect, and self-control, and is not interpreted substantively.

## Supplementary Table S4. Residual and influence diagnostics for the fully adjusted models

| **Model** | **Residual skew** | **Residual excess kurtosis** | **Max Cook’s D** | **Cases with Cook’s D > 4/n** |
| --- | --- | --- | --- | --- |
| IGD Model C | 0.49 | 0.71 | 0.074 | 27 |
| PSMU Model C | −0.24 | 0.17 | 0.061 | 27 |

Note. No case approached conventional thresholds of concern (all Cook’s D < 0.08 against a threshold of 1.0; the 4/n heuristic flags 7.5% of cases, consistent with expectation). Residual distributions were approximately normal by visual inspection, with mild positive skew for the IGD model.

## **Supplementary Table S5. Contextual associations of family-related factors and basic psychological need satisfaction with IGD symptoms and PSMU (n = 358)**

| **Contextual variable** | **IGD β (95% CI)** | **p** | **PSMU β (95% CI)** | **p** |
| --- | --- | --- | --- | --- |
| Parental emotional warmth | −.051 [−.221, .119] | .553 | .008 [−.169, .185] | .928 |
| Parental autonomy support | −.060 [−.300, .180] | .623 | −.029 [−.278, .220] | .819 |
| Parent–child relationship quality | .064 [−.159, .287] | .573 | −.146 [−.377, .085] | .216 |
| Basic psychological need satisfaction | −.062 [−.244, .121] | .507 | .067 [−.123, .256] | .489 |

Note. The four contextual variables were entered simultaneously in separate ordinary least-squares models for IGD symptoms and PSMU, with sex and age included as covariates. Outcomes and the four contextual variables were z-standardized; sex and age were entered using their original coding and units. IGD model: adjusted R² = .077, F(6, 351) = 5.97, p < .001. PSMU model: adjusted R² = .005, F(6, 351) = 1.29, p = .263. None of the four focal contextual coefficients was statistically significant in either model.
